# Recent Advances in 3D Printing of Two-Dimensional Materials and Devices

**DOI:** 10.3390/ma19204361

**Published:** 2026-10-16

**Authors:** Wei-Min Shao, Yun-Zhuo Zhang, Shu-Qi Wang, Jia-Hao Li, Jia-Jun Zhao, Ya-Ming Wang, Yong-Chun Zou, Jia-Hu Ouyang

**Affiliations:** School of Materials Science and Engineering, Harbin Institute of Technology, Harbin 150001, China; 25s109156@stu.hit.edu.cn (W.-M.S.); 21b909010@stu.hit.edu.cn (Y.-Z.Z.); 24s109246@stu.hit.edu.cn (J.-H.L.); 24s009048@stu.hit.edu.cn (J.-J.Z.); wangyaming@hit.edu.cn (Y.-M.W.); zouyongchun@hit.edu.cn (Y.-C.Z.)

**Keywords:** two-dimensional materials, 3D printing, functional inks, energy storage, biomedical, devices

## Abstract

Two-dimensional (2D) materials offer opportunities for constructing functional devices; however, integrating them into macroscopic structures requires precise control over nanosheet dispersion, processing parameters, and structural design. This review summarizes research on the fabrication of 3D structures using nanosheet-based inks and composite feedstocks—including graphene and its derivatives, MXenes, transition metal dichalcogenides, hexagonal boron nitride, and black phosphorus. Studies indicate that material performance is determined by the combined effects of feedstock composition, printing conditions, structural design, and post-processing techniques. Direct Ink Writing (DIW) is a particularly suitable fabrication technique for various porous lattices and self-supporting structures where nanosheets form the primary functional network. High-concentration aqueous MXene inks and rheologically tuned graphene oxide-based inks serve as typical feedstocks, provided they exhibit favorable shear-thinning behavior, shape retention, and structural recovery capabilities. Regarding polymer-based composite components, Fused Deposition Modeling (FDM) is applicable to thermoplastic feedstocks loaded with 2D materials, whereas Stereolithography (SLA) and Digital Light Processing (DLP) utilize nanosheet-loaded photocurable resins to fabricate composites with intricate features, albeit with applications constrained by light attenuation and curing characteristics. Applications in sensing, energy storage devices, and biomedical scaffolds demonstrate the immense potential of these methods, while printed optoelectronic devices are discussed as a complementary case of device integration. Nevertheless, challenges such as material instability, trade-offs between resolution and scale, post-processing shrinkage, and incomplete biosafety assessments remain significant barriers to practical application, highlighting the need to develop reproducible processing techniques and conduct validation studies focused on real-world use.

## 1. Introduction

Two-dimensional (2D) materials, characterized by their atomic-scale thickness and unique planar structures, have revolutionized materials science since the isolation of graphene in 2004 [1]. These materials, including graphene, transition metal dichalcogenides (TMDs) such as MoS_2_ and WS_2_, MXenes, hexagonal boron nitride (*h*-BN), and others, exhibit exceptional properties like high electrical conductivity, mechanical strength, tunable bandgaps, and chemical stability, making them ideal for applications in electronics, energy storage, biomedicine, and sensing [2,3,4,5]. However, their inherent two-dimensionality limits their scalability and integration into three-dimensional (3D) architectures, where enhanced surface area, porosity, and structural complexity are often required for practical devices [6].

Additive manufacturing, commonly known as 3D printing, has emerged as a transformative technology for fabricating complex, customized structures with precise control over geometry and composition [7,8].Techniques such as direct ink writing (DIW), fused deposition modeling (FDM), stereolithography (SLA), digital light processing (DLP), and selective laser sintering (SLS) enable layer-by-layer construction from digital models, offering advantages in cost-efficiency, material versatility, and rapid prototyping [9]. In the context of 2D materials, 3D printing addresses key limitations by assembling these nanosheets into hierarchical 3D frameworks, such as aerogels, scaffolds, and lattices, thereby unlocking enhanced functionalities like improved electrochemical performance and mechanical resilience [10].

MXenes have attracted widespread attention in the field of 3D printing. Regarding Direct Ink Writing (DIW) technology, the widely studied Ti_3_C_2_Tₓ-type MXenes possess hydrophilic surfaces that facilitate the formulation of aqueous inks, while their suitable rheological properties ensure successful extrusion and shape retention after deposition [7]. In Fused Deposition Modeling (FDM), MXenes are incorporated into thermoplastic composite filaments; their printability depends on filler dispersion, melt rheology, thermal stability, and reliable filament feeding. Electrodeposition and laser-assisted methods facilitate phase-tunable growth of TMDs, while aligned stacking enables heterostructures like MoS_2_/graphene with atomic precision [11]. MXenes, in particular, stand out for additive-free inks in DIW, yielding micro-supercapacitors with very high areal capacitance of up to ∼1035 mF·cm^–2^ [7].

These developments have expanded applications across fields. In energy storage, 3D-printed MXene-based aerogels and composites exhibit high volumetric capacitance and rate performance [12]. In biomedicine, 2D materials like graphene oxide (GO) and MXenes integrated into 3D scaffolds promote osteogenic differentiation for bone regeneration and photothermal therapy for tumor ablation, with high biocompatibility in vivo [13]. Electronics benefit from scalable synthesis and printing of TMDs and graphene for sensors and transistors with enhanced sensitivity [14]. Recent progress includes DIW of additive-free MXene inks for flexible devices and hybrid aerogels for multifunctional applications [15].

Several review articles have laid a significant foundation in this field. Gómez et al. reviewed major 2D material systems, printing techniques based on extrusion and photocuring, inkjet printing, and post-functionalization treatments, while also exploring applications in sensing, biomedicine, energy storage, and optoelectronics [16]. Their review addressed ink rheology, processing-induced alignment, and optical constraints, and summarized data regarding formulations and properties. More recently, Khond and More examined MXene-based 3D printing technologies, focusing on applications in medical devices, textiles, electromagnetic shielding, sensors, and energy storage [6]. Although they outlined other 2D materials and cited examples involving DLP technology and the controlled assembly of nanosheets, their detailed analysis of applications remained primarily focused on MXene systems. These studies provide a basis for more comprehensive comparative analyses—specifically, those that holistically consider the relationships between material selection for different 2D material systems, specific process requirements, structural evolution, and device performance.

Building upon existing reviews and incorporating recent research progress, this article discusses the printability of two-dimensional (2D) materials, the relationships between processing and structural performance, and key challenges in their application. First, it summarizes the material characteristics, preparation methods, and formulation requirements for graphene and its derivatives, TMDs, MXenes, *h*-BN, and BP, while examining how surface chemistry, dispersion behavior, and stability influence printability. Next, it focuses on analyzing rheological control and shape retention in Direct Ink Writing (DIW), melt processing and interlayer fusion in Fused Deposition Modeling (FDM), and light attenuation and curing behavior in SLA/DLP, alongside an introduction to inkjet printing and related deposition techniques. Based on this, and drawing on representative studies regarding sensors, supercapacitors, lithium-ion batteries, biomedical scaffolds, and photodetectors, the article discusses the links between functional performance of printed structures and devices and factors such as flake orientation, pore structure, interlayer interfaces, and conductive networks. Finally, it outlines challenges including material stability, printing limitations, biosafety, ink diversity, trade-offs between resolution and scale, and post-processing shrinkage. Compared to the earlier review by Gómez et al., this article incorporates subsequent research developments, placing particular emphasis on process-specific requirements and the structure-property relationship; furthermore, unlike the MXene-focused review by Khond and More, this article extends the discussion of material-process compatibility to a broader range of 2D materials. Thus, this article contributes a comprehensive analysis of the interconnections among materials, processing, structure, and applications in light of recent research.

**Figure 1:**
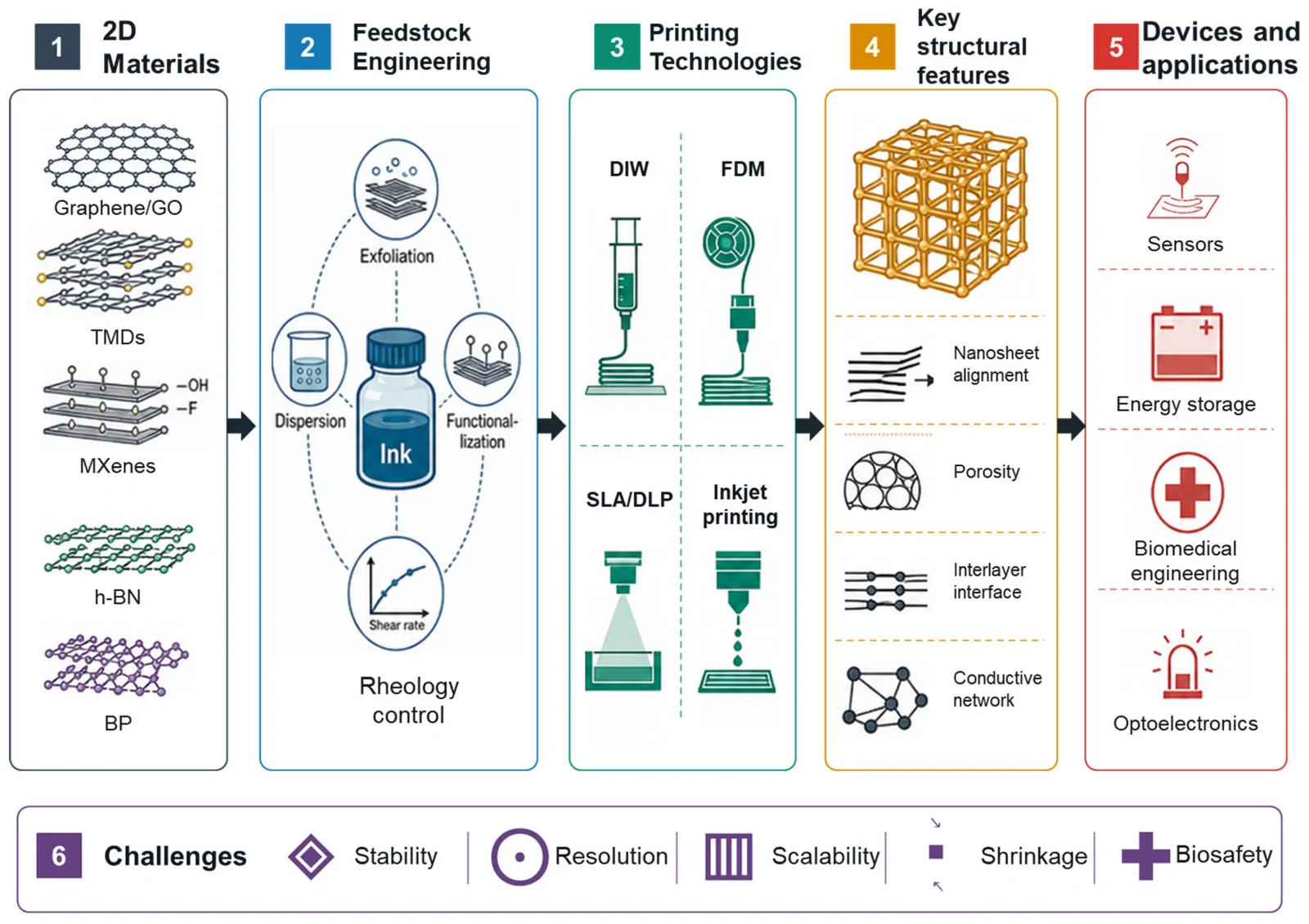
Overall framework for 3D printing of two-dimensional materials and devices.

Figure 1 outlines the entire process, ranging from 2D material selection and feedstock engineering to printing, structural modulation, and functional applications. Various 3D printing technologies are currently employed, including direct ink writing (DIW), fused deposition modeling (FDM), and stereolithography/digital light processing (SLA/DLP), while inkjet printing can also be used to achieve planar patterning. The figure highlights key factors such as nanosheet orientation, porosity, interlaminar interfaces, and conductive networks, while also identifying challenges related to stability, resolution, scalability, shrinkage, and biosafety.

## 2. Types and Properties of Two-Dimensional Materials

Two-dimensional (2D) materials are crystalline substances confined to atomic-scale thickness, leading to quantum confinement effects that manifest in exceptional electronic, optical, mechanical, and thermal properties distinct from their bulk counterparts [17]. These attributes, including high surface-to-volume ratios, tunable bandgaps, and van der Waals interlayer interactions, render them highly compatible with additive manufacturing processes such as 3D printing for hierarchical architectures [2]. Common synthesis routes encompass top-down methods and bottom-up approaches [18]. This section delineates principal 2D material classes, emphasizing structures, synthesis, and properties pertinent to 3D printing applications.

### 2.1. Graphene and Its Derivatives

Graphene comprises a single layer of sp^2^-hybridized carbon atoms in a hexagonal lattice, exhibiting extraordinary electrical conductivity, mechanical robustness, and thermal conductivity [19,20,21]. Graphene, graphene oxide (GO) and reduced graphene oxide (rGO) can be used to construct conductive and mechanically enhanced network. Primary synthesis involves mechanical exfoliation, Chemical Vapor Deposition (CVD) on metallic substrates or Liquid-Phase Exfoliation (LPE) from graphite [22]. Oxidized derivatives like graphene oxide (GO) and reduced GO (rGO) incorporate hydrophilic functional groups, enhancing solvent dispersibility for ink formulation while moderately reducing conductivity [23]. Graphene’s conductive percolation networks in composites augment electrochemical performance, making it ideal for 3D-printed energy devices.

### 2.2. Transition Metal Dichalcogenides (TMDs)

TMDs follow the formula MX_2_ (M = transition metal, e.g., Mo, W; X = chalcogen, e.g., S, Se), featuring layer-dependent electronic transitions: indirect bandgap in bulk to direct bandgap in monolayers [4]. They offer tunable optoelectronic properties, moderate mobility, and phase polymorphism [24]. TMDs such as MoS_2_ and WS_2_ possess multiple functions including semiconductor, catalysis, lubrication and optoelectronic. Synthesis predominantly utilizes CVD for uniform films or LPE in solvents like N-Methyl-2-pyrrolidone (NMP) for high-throughput nanosheets [25]. In 3D printing, TMDs such as MoS_2_ contribute catalytic sites and lubricity to multifunctional inks.

### 2.3. MXenes

MXenes, formulated as M_n + 1_X_n_T_x_ (M = early transition metal; X = C/N; T_x_ = surface terminations, e.g., -O, -OH, -F), derive from selective etching of MAX phases [26]. They display metallic conductivity [27], high volumetric capacitance [28]. MXenes are highly attractive in the field of water-based printing, thanks to the hydrophilicity imparted by their surface functional groups and their shear-thinning properties that vary with concentration. Its main limitation lies in its poor chemical stability: oxidation can alter the material’s conductivity, lamellar morphology, and electrochemical behavior. Monolayer Young’s modulus reaches ~0.33 TPa [3], with shear-thinning rheology ideal for direct ink writing (DIW) [29]. Delamination via intercalants yields large flakes, positioning MXenes as premier candidates for 3D-printed EMI shielding and energy storage.

### 2.4. Hexagonal Boron Nitride (h-BN)

*h*-BN, an isostructural analog to graphene with alternating B and N atoms, functions as a wide-bandgap insulator with superior in-plane thermal conductivity and chemical inertness [30,31]. Mechanical strength and dielectric properties suit it for van der Waals heterostructures [32,33]. Synthesis includes CVD on catalytic substrates or LPE in polar solvents [34]. In composites, *h*-BN enhances thermal management while preserving electrical insulation, critical for 3D-printed heat-dissipating electronics.

### 2.5. Black Phosphorus (BP)

BP exhibits a puckered orthorhombic structure with layer-dependent direct bandgap, enabling mid-infrared photodetection [35]. Anisotropic mobility reaches ~1000 cm^2^ V^−1^ s^−1^ along the armchair direction, though ambient oxidation necessitates passivation [36]. Exfoliation typically occurs mechanically or via Liquid-Phase Exfoliation (LPE) in deoxygenated solvents [37]. BP integrates into bioinks for photothermal applications, leveraging high Near-Infrared (NIR) extinction.

### 2.6. Other 2D Materials

Emerging classes encompass layered double hydroxides (LDHs), transition metal oxides, and 2D perovskites exhibiting strong excitonic effects [38,39]. These broaden ink versatility for redox-active or porous 3D structures. The multifaceted properties of 2D materials arise from atomic confinement and engineering via synthesis or functionalization, enabling tailored integration into 3D-printed multifunctional devices.

## 3. Application of 3D Printing Techniques to Two-Dimensional Materials

The convergence of additive manufacturing (AM), commonly referred to as 3D printing, with two-dimensional (2D) materials has enabled the fabrication of complex, hierarchical architectures that transcend the limitations of planar configurations inherent to nanosheets [40,41]. By assembling 2D building blocks—such as graphene, transition metal dichalcogenides (TMDs), MXenes, hexagonal boron nitride (*h*-BN), and black phosphorus (BP)—into three-dimensional (3D) macrostructures, enhanced properties including high specific surface area, tunable porosity, and multifunctionality (e.g., combined electrical conductivity and mechanical resilience) are realized [2,10]. This section delineates the requisite characteristics of 2D materials for printability, categorizes applicable 3D printing modalities with detailed subprocess descriptions, and elucidates the mutual interfacial requirements and challenges governing material-process compatibility.

**Figure 2:**
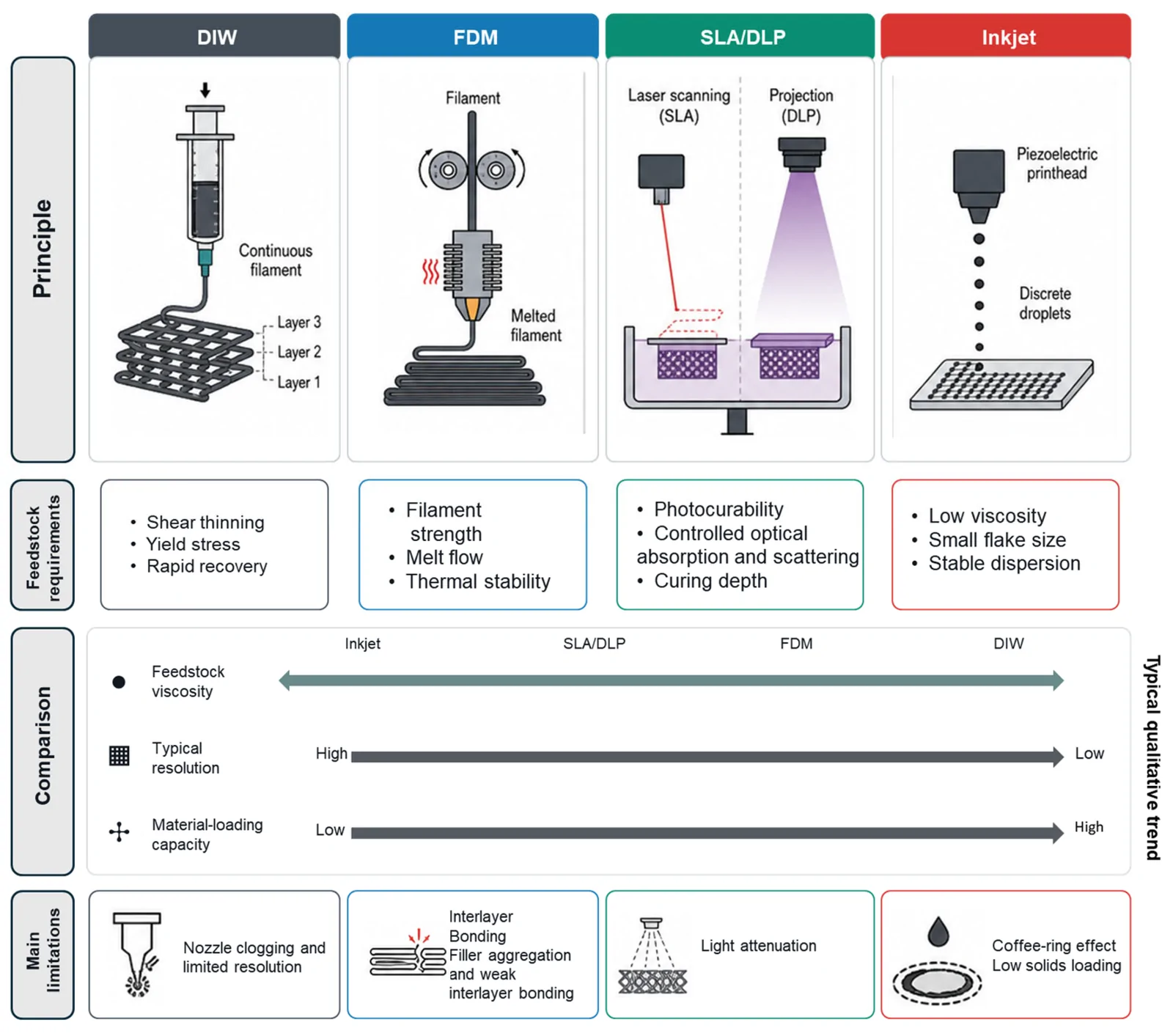
Operating principles, feedstock requirements, and limitations of major printing technologies for 2D materials.

The operating principles, feedstock requirements, characteristic advantages, and major limitations of the four representative printing routes are compared in Figure 2.

### 3.1. Characteristics of 2D Materials Enabling 3D Printability

Printability of 2D materials is predicated on their colloidal stability, surface chemistry, and rheological modulation in ink formulations [7]. High aspect ratios and lateral dimensions facilitate percolation networks at low loadings, yielding conductive pathways post-printing [42]. Hydrophilicity, imparted by surface terminations (e.g., -O, -OH, -F in MXenes), enables additive-free aqueous dispersions with concentrations exceeding 200 mg mL^−1^, exhibiting pronounced shear-thinning behavior and viscoelastic properties [43,44].

In contrast, for materials with limited dispersibility in water (such as pristine graphene and certain transition metal dichalcogenides), it may be necessary to employ suitable solvents, surface functionalization, or dispersing agents to mitigate aggregation and restacking, thereby achieving a higher absolute Zeta potential and generating electrostatic repulsion [23]. Zeta potential helps assess the contribution of electrostatic repulsion, yet there is no universal threshold applicable to all 2D material inks, as its value depends on various factors such as the dispersion medium, pH, ionic strength, nanosheet characteristics, and additives. Mechanical integrity, characterized by in-plane stiffness, ensures structural fidelity during deposition and post-processing, while thermal/chemical stability resists degradation in elevated-temperature or photopolymerization environments [3,20]. Biocompatibility and stimuli-responsiveness (e.g., NIR absorption in BP for photothermal effects) further broaden applicability in biomedical inks [45].

### 3.2. Extrusion-Based Printing Techniques

Extrusion-based printing technology is one of the most widely studied methods for fabricating three-dimensional structures using two-dimensional materials.

#### 3.2.1. Direct Ink Writing (DIW)

Direct ink writing (DIW), also known as robocasting, is one of the most widely investigated extrusion-based printing techniques for fabricating three-dimensional structures from 2D materials [46]. It employs pneumatic, piston-, or screw-driven extrusion through a nozzle to deposit continuous filaments in a layer-by-layer manner. A key advantage of DIW is its broad compatibility with highly concentrated, particle- or nanosheet-loaded inks, enabling the fabrication of complex architectural structures. Unlike printing techniques that require low-viscosity inks or photoresponsive feedstocks, DIW relies primarily on the rheological characteristics of the ink, making it applicable to a broad range of 2D-material systems, including graphene, graphene oxide (GO), MXenes, and other nanosheet-based suspensions. By tailoring the ink formulation and rheological behavior, continuous extrusion and sufficient structural retention after deposition can be achieved, thereby providing considerable flexibility in the design and fabrication of 3D 2D-material architectures [47,48,49].

For 2D-material inks, printability is strongly governed by the balance between flowability during extrusion and shape retention after deposition. A suitable DIW ink generally exhibits shear-thinning behavior, whereby its apparent viscosity decreases under the high shear conditions generated inside the nozzle, thereby facilitating continuous extrusion under an applied pressure. At the same time, sufficient yield stress and structural recovery are required to maintain the deposited filament geometry after extrusion and to prevent excessive spreading or collapse of the printed layers. Thus, rather than relying on a single rheological parameter, the printability of 2D-material inks is commonly evaluated using yield stress, shear viscosity, storage modulus (G′), loss modulus (G″), and thixotropic or structural recovery behavior. In particular, a relatively high low-shear viscosity or yield stress can contribute to filament stability, whereas rapid shear-thinning facilitates flow through the nozzle. Importantly, these characteristics should not be regarded as universal numerical thresholds because the appropriate rheological window depends strongly on nanosheet concentration, lateral size and aspect ratio, solvent composition, nozzle diameter, extrusion pressure, printing speed, and the target architecture [29].

The intrinsic characteristics of 2D nanosheets can strongly influence these rheological properties. Increasing nanosheet concentration generally increases interparticle interactions and can promote the formation of a percolated or liquid-crystalline network, resulting in increased viscosity and elastic response. At the same time, nanosheet aspect ratio and surface chemistry affect dispersion stability and intersheet interactions, thereby determining whether a stable printable suspension or an aggregated system is obtained. For example, rheological investigations of Ti_3_C_2_T_x_ MXene dispersions demonstrated pronounced concentration-dependent rheological behavior, providing an important basis for designing MXene inks for extrusion-based processing. Similarly, GO has been demonstrated to act not only as a functional 2D material but also as a rheological modifier, enabling aqueous suspensions with shear-thinning behavior and rapid recovery of their elastic properties for robocasting [29,50]. These observations indicate that the formulation of printable 2D-material inks requires a balance between sufficiently strong interparticle interactions for structural retention and sufficiently low flow resistance during extrusion.

Recent research has further elucidated the relationship between the rheological properties of MXene inks, nanosheet alignment, and electrode performance. Cao et al. developed a liquid-crystalline MXene ink for micro-supercapacitors, enabling the 3D printing of thick electrodes free from restacking [51]. This approach combines printable viscoelastic characteristics with controllable nanosheet alignment, allowing for the fabrication of thick electrodes with customizable dimensions. The study clarifies how ink formulation modulates deposition behavior and the internal structure of printed electrodes, establishing a link between printability and electrochemical functionality.

To fabricate porous cellular structures, digital models are converted into deposition paths that dictate filament placement, spacing, and interlayer arrangement. During printing, the nozzle traverses these paths while extruding ink at a controlled flow rate. The dimensions of the deposited filaments are determined by the nozzle diameter, extrusion flow rate, printing speed, and the gap between the nozzle and the substrate. Coordinating these parameters is crucial for maintaining continuous deposition and accurately replicating the intended geometry. Arrays of spaced filaments deposited in alternating orientations create interconnected channels, whereas contour-based paths form the walls of other cellular structures. Consequently, the macroscopic pore geometry is primarily established through programmed material deposition rather than relying solely on nanosheet assembly.

Subsequent layers are deposited at predetermined locations, contacting previously deposited filaments to ensure structural continuity. A balance must be struck between shape retention and inter-filament bonding strength: excessive spreading can narrow or close designed pores, while insufficient contact may lead to structural discontinuities or weak interfacial bonding. When filaments span voids, their resistance to sagging depends on ink properties, the unsupported span length, and the time required for structural stabilization. Depending on the formulation, the printed structures are subsequently stabilized through drying, gelation, cross-linking, or other curing processes. It is important to distinguish the macroscopic channels defined by the printing paths from the micropores formed within the filaments during freeze-drying or other post-processing steps. Thus, the final structural morphology reflects both the outcome of programmed deposition and subsequent structural evolution (including shrinkage or deformation).

For functional porous or lattice structures, the interplay between ink rheology, the deposition process, and post-processing techniques is particularly critical. For instance, MXene-based inks have been utilized to fabricate 3D self-supporting structures featuring interconnected conductive networks. Typical structures exhibit electrical conductivities exceeding 10^4^ S m^−1^ and compressive moduli surpassing 1 MPa, demonstrating the potential of combining engineered structures with functional nanosheet networks. However, these values depend on material composition, structural design, and post-processing techniques and thus should not be regarded as intrinsic material properties of MXene inks [11,16].

Direct Ink Writing (DIW) technology also influences the spatial arrangement of anisotropic nanosheets. During extrusion, shear and extensional flows can promote the alignment of nanosheets, with the final orientation state determined by nozzle geometry, flow conditions, and ink composition. This orientation, combined with the printing direction and hierarchical arrangement, dictates the material’s anisotropic electrical, mechanical, thermal, or electrochemical behavior. Research on graphene-based DIW systems and shear-exfoliated graphene/hexagonal boron nitride (*h*-BN) formulations indicates that constructing ordered structures and leveraging flow-induced nanosheet alignment are effective strategies for tuning functional properties [14,52]. Consequently, nozzle-induced flow affects both material delivery and the internal microstructure of the deposited filaments.

This process necessitates trade-offs across several aspects. While increasing the loading of 2D materials enhances functional content and network connectivity, excessive loading can raise extrusion resistance and aggravate agglomeration, potentially leading to nozzle clogging or discontinuous deposition; conversely, lower loading facilitates extrusion but may compromise the density and connectivity of the functional network. Variations in printing speed also alter the interplay between material delivery, filament dimensions, and structural recovery. Therefore, the successful fabrication of such structures requires the synergistic optimization of material concentration, nanosheet size, dispersion state, rheological properties, nozzle geometry, extrusion conditions, and deposition paths.

Furthermore, DIW technology enables the fabrication of multi-material and spatially heterogeneous structures. By employing multiple nozzles or sequentially switching inks, conductive, insulating, active, and structural materials can be deposited at predetermined locations within a single structure. Such strategies enable the development of integrated devices for energy storage, sensing, and multifunctional applications [16]. However, to ensure structural continuity and avoid defects, the inks used must be mutually compatible in terms of rheological properties, interfacial interactions, curing behavior, and post-processing requirements. Depending on the formulation, these structures may manifest as functional networks dominated by nanosheets or as matrix composites containing two-dimensional fillers; specific classification depends on the actual material composition.

Overall, Direct Ink Writing (DIW) technology synergistically integrates ink rheological properties with controlled filament deposition, nanosheet alignment, and 3D structural design. When the ink can maintain its prescribed geometry during deposition and subsequent processing, the technology is particularly well-suited for fabricating porous, cellular, lattice, and multi-material structures. However, the achievable feature sizes and structural fidelity are constrained by factors such as nozzle size, filament spreading, unsupported spans, inter-filament bonding, and post-processing deformation. These considerations differ from those associated with FDM (Fused Deposition Modeling)—discussed later—in which thermoplastic feedstock is deposited in a softened or molten state and solidifies primarily through cooling.

#### 3.2.2. Fused Deposition Modeling (FDM)

Fused Deposition Modeling (FDM), also known as Fused Filament Fabrication (FFF), is a material extrusion-based additive manufacturing technique. In this process, a thermoplastic filament is heated above its processing temperature, extruded through a heated nozzle, and deposited layer-by-layer to construct a three-dimensional object. When applied to 2D materials, FDM typically employs thermoplastic composites containing fillers such as graphene, graphene nanoplatelets (GNPs), graphene oxide (GO), MXenes, or other nanosheet-based materials. Filaments containing these fillers soften or melt within the heated nozzle and subsequently solidify primarily through cooling [6]. Unlike Direct Ink Writing (DIW), which primarily deals with liquid or paste-like inks, FDM requires the incorporation of 2D materials into a thermoplastic feedstock that possesses sufficient filament strength, melt processability, and thermal stability. Consequently, the primary processing considerations shift from ink rheology and post-extrusion structural recovery to filament feeding, melt flow behavior, thermal stability, and interlayer fusion. The incorporation of 2D materials can significantly alter the melt flow behavior and processing characteristics of thermoplastic filaments. Due to the high specific surface area of graphene-based nanosheets and their strong interfacial interactions with polymer chains, increasing filler content typically raises melt viscosity and may reduce flowability during extrusion. Furthermore, inadequate dispersion can lead to agglomeration, thereby increasing extrusion resistance, promoting nozzle clogging, and introducing defects into the printed structure. Therefore, FDM processing requires striking an appropriate balance among 2D material concentration, nanosheet dispersion, polymer-filler interactions, melt viscosity, and processing temperature [53]. Unlike DIW, where shear thinning and structural recovery of the ink are critical for printability, the processability of FDM feedstock depends primarily on the thermal softening or melting behavior of the polymer matrix and the resulting melt rheology. Thus, the filler loading should be optimized within the processing window of the specific material.

During the FDM process, the polymer melt undergoes shear and extensional deformation as it passes through the nozzle. These flow fields can induce the preferential alignment of anisotropic 2D nanosheets along the extrusion direction. While this alignment facilitates the formation of directional pathways for electrical and thermal transport—thereby enhancing properties along the printing direction—it also increases the anisotropy of the final component. Consequently, parameters such as nozzle diameter, extrusion temperature, printing speed, raster orientation, and layer stacking sequence influence nanosheet orientation and the resulting structure–property relationships [54,55]. This characteristic distinguishes FDM-processed 2D material composites from randomly dispersed bulk composites, as the printing process itself can alter the spatial distribution and orientation of functional fillers.

Interlayer bonding is another critical factor determining the performance of FDM-printed composites. Following deposition, adjacent thermoplastic filaments must undergo sufficient interfacial contact, polymer chain diffusion, and solidification to form mechanically continuous layers. The incorporation of 2D fillers alters polymer fluidity, crystallization behavior, melt viscosity, and heat transfer, thereby influencing the formation of interlayer interfaces. Insufficient nozzle or build-platform temperatures and excessive printing speeds can restrict interlayer diffusion and promote the formation of voids or weak interfaces, whereas appropriate thermal conditions facilitate interlayer fusion. Thus, the mechanical and functional properties of FDM-printed 2D material composites depend not only on filler content and dispersion but also on the thermal history and deposition conditions of the printed layers.

The thermal history during the FDM process is particularly significant for chemically or structurally sensitive 2D materials. For instance, graphene oxide (GO) may undergo partial thermal reduction during melt processing, leading to changes in its oxygen-containing functional groups, electrical conductivity, and interaction with the polymer matrix [56]. Although this transformation can enhance electrical transport by increasing the degree of reduction, it simultaneously alters dispersion characteristics and interfacial interactions. This example illustrates that the thermal processing window for FDM must be selected based on the stability of both the polymer matrix and the added 2D materials.

The FDM process involves a trade-off between functional filler content and processability. Increasing the concentration of graphene or other 2D fillers raises the probability of forming interconnected filler networks, thereby enhancing electrical conductivity, thermal conductivity, stiffness, and other functional properties. However, excessive filler loading leads to increased melt viscosity, reduced filament flexibility and feeding reliability, filler agglomeration, and weakened interlaminar bonding strength. For instance, researchers have successfully produced ABS filaments containing 4 wt% graphene nanoplatelets (xGnP) via FDM; the resulting printed composites exhibited higher elastic modulus, dynamic storage modulus, and superior thermal stability, although the addition of graphene also reduced the material’s fracture stress and fracture strain [55]. Similar studies on polymer/graphene systems indicate that the optimal filler concentration is not a universal constant but depends heavily on the polymer matrix, filler morphology, dispersion state, and target properties [53,57].

Beyond graphene systems, the application of FDM technology has expanded to other two-dimensional (2D) materials. For example, material extrusion processes can be used to fabricate thermoplastic composites containing MXenes, enabling the creation of 3D structures that possess both functional and stimulus-responsive characteristics. In a representative study, researchers used material extrusion to process a TPU/PLA composite containing 2 wt% MXene, producing 4D-printed structures that exhibited thermo-responsive shape-memory behavior. Such studies demonstrate that FDM offers a convenient pathway for integrating 2D materials into thermoplastic structures, while simultaneously leveraging the distinct shape-memory, conductive, mechanical, or other functional properties of both the polymer matrix and the nanoplatelets [58].

Overall, the primary advantages of using FDM technology for 2D material-based 3D printing include compatibility with thermoplastics, relatively simple feedstock processing, minimal material waste, and the ability to manufacture components with robust mechanical properties and complex geometries. However, the application of this technology is constrained by the need to process 2D materials into melt-processable filaments or pellets, as well as by the inherent thermal stability limits of the polymer matrix and the nanoplatelets themselves. Furthermore, issues such as nozzle-induced orientation effects, filler agglomeration, thermally induced reduction of graphene oxide (GO), interlaminar voids, and anisotropic bonding may cause the material to exhibit direction-dependent mechanical, electrical, and thermal properties.

### 3.3. Photopolymerization-Based Printing Techniques

Vat photopolymerization enables the fabrication of complex structures with relatively high spatial resolution.

#### 3.3.1. Stereolithography (SLA)

Stereolithography (SLA) is a representative vat photopolymerization (VPP) additive manufacturing technology that constructs three-dimensional structures by selectively curing photosensitive resin using an ultraviolet (UV) or visible light source in a layer-by-layer manner. In conventional laser-scanning SLA, a focused light beam scans the resin surface along a preset cross-sectional path, triggering photopolymerization in the illuminated regions; once a layer is cured, the build platform repositions according to the set layer thickness to allow for the exposure and curing of the subsequent layer. SLA offers high forming precision and surface quality; its geometric accuracy is collectively influenced by factors such as laser power, scanning speed, layer thickness, scan spacing, as well as the resin’s light penetration depth and critical exposure dosage [59,60]. Consequently, compared to nozzle-extrusion-based techniques like DIW and FDM, SLA is better suited for fabricating 3D structures characterized by complex geometries and fine feature sizes.

Fabricating 2D-material-based composite structures via SLA typically requires the uniform dispersion of 2D nanosheets within the photocurable resin. A standard photocurable resin system comprises polymerizable monomers or oligomers, photoinitiators, and necessary reactive diluents alongside other functional additives. Research has demonstrated that graphene, graphene oxide (GO), and their functionalized derivatives can be incorporated into acrylate-based photosensitive resins to fabricate 2D-material/polymer 3D composite structures using SLA [61,62,63]. For instance, Korhonen et al. utilized a photocurable resin/GO system to fabricate graphene-based components with predefined 3D architectures, thereby validating the feasibility of using SLA to construct 3D structures from 2D carbon materials [61]. Furthermore, Manapat et al. incorporated small amounts of GO into commercial SLA resins and investigated the effects of GO content and subsequent heat treatment on the mechanical properties and thermal stability of the printed composites, further demonstrating the effective integration of GO with the photocurable resin system [62]. In recent years, functionalized GO has also been employed to enhance dispersion stability within acrylate resins and to enable SLA fabrication at high filler loadings [63].

Unlike DIW, which relies primarily on ink rheology, and FDM, which depends on thermoplastic melt flow, the SLA fabrication process is collectively influenced by factors such as resin viscosity, nanosheet dispersion stability, optical attenuation, photoinitiator efficiency, and photopolymerization kinetics [59,60]. Resin viscosity directly affects the recoating process following the completion of an exposure layer, while the incorporation of 2D nanosheets can further alter the system’s rheological properties. For instance, increasing GO content raises the viscosity of the photosensitive resin and—at higher loadings—induces non-Newtonian behavior and nanosheet agglomeration, thereby impacting resin spreading and fabrication stability [64]. Consequently, for 2D material/SLA systems, maintaining appropriate resin viscosity and stable nanosheet dispersion is crucial for ensuring continuous fabrication.

The optical interaction between 2D materials and incident light is a critical limiting factor in the SLA fabrication of 2D material composite systems. Many 2D materials with strong light-absorbing capabilities can significantly alter light propagation within the photosensitive resin; simultaneously, the refractive index mismatch between nanosheets and the resin, along with particle size, morphology, and spatial distribution, can induce light scattering, causing the incident light energy to attenuate with increasing propagation depth. In photocurable systems containing solid fillers, both light absorption and scattering reduce the effective light intensity, thereby affecting the cure depth and the spatial distribution of the exposed region [60]. Consequently, excessive 2D material content or significant agglomeration can lead to reduced effective cure depth, non-uniform local exposure, and compromised dimensional accuracy. For 2D material/SLA systems, parameters such as nanosheet concentration, flake size and dispersion state, resin layer thickness, exposure dosage, photoinitiator concentration, and photocuring wavelength must be comprehensively regulated to establish a stable photocuring window.

It is worth noting that the influence of 2D nanosheets on the photopolymerization process extends beyond light absorption and scattering; their surface chemical properties can also alter interfacial interactions between the nanosheets and resin components, as well as the polymerization reaction itself. For instance, oxygen-containing functional groups on the GO surface can form interactions—such as hydrogen bonding—with the polymer matrix, thereby improving interfacial adhesion between the filler and the polymer; conversely, as a carbonaceous filler with strong optical absorption, GO may also inhibit the photopolymerization process. Studies have shown that increasing GO content reduces the degree of gelation in photosensitive resins, suggesting that GO may restrict polymer chain mobility and hinder further polymerization [65]. Thus, the role of 2D materials in photocuring systems is distinctly dual-natured; their ultimate impact depends on factors such as filler surface chemistry, content, dispersion state, and interactions with photoinitiators and resin components. Rather than simply increasing the loading of 2D materials, enhancing interfacial compatibility between the 2D material and the resin—through surface functionalization and dispersion control—may be a key strategy for broadening the SLA processing window [65].

Another significant advantage of SLA is its ability to directly fabricate 3D structures with complex geometries—including lattice structures, porous architectures, and designs featuring intricate details—from digital models. For 2D material composite systems, this high geometric fidelity enables the construction of complex macro-scale structures while simultaneously incorporating 2D materials into the polymer network, thereby integrating structural design with functional materials. Research has already demonstrated the use of SLA to fabricate GO/polymer and functionalized GO/acrylate composites with predefined 3D geometries [63]. However, unlike the pronounced flake alignment observed in DIW and FDM processes—driven by shear and extensional flows within the nozzle—2D nanosheets in SLA remain primarily suspended in the photosensitive resin; their final orientation is influenced more by the initial dispersion state, resin flow, curing shrinkage, and post-processing steps.

Following the printing process, SLA parts typically require cleaning to remove residual resin from surfaces and uncured regions. They also undergo post-curing—using UV or visible light, depending on the specific resin system—to enhance polymerization conversion and final mechanical stability [60]. For 2D material/SLA composite systems, this post-processing stage can further alter the interfacial structure between the nanosheets and the polymer matrix, as well as the material’s overall properties. For example, following heat treatment, the polymer network, GO structure, and filler–matrix interface of GO/SLA composites may undergo changes, thereby altering their mechanical and electrical properties [62]. Consequently, optimizing the performance of 2D material-based SLA systems requires considering not only the printing stage but also the cleaning, post-curing, and—where necessary—heat treatment processes as part of the overall processing window.

#### 3.3.2. Digital Light Processing (DLP)

Digital Light Processing (DLP) is a significant vat photopolymerization technique used to fabricate three-dimensional structures from photosensitive materials. Unlike Stereolithography (SLA), which selectively cures resin using a scanning laser beam, DLP employs a digital projection system—typically based on a Digital Micromirror Device (DMD)—to project an entire two-dimensional cross-sectional pattern onto the resin surface. Consequently, an entire layer can be polymerized in a single exposure step, eliminating the need for sequential, point-by-point scanning. This parallel exposure mechanism grants DLP a distinct advantage in printing efficiency while maintaining the capability to fabricate complex, high-resolution structures [59,66].

For photocurable resins containing 2D materials, the fundamental printing mechanism of DLP relies on the interaction between the projected light and the nanosheet-loaded resin. Similar to SLA, 2D materials can alter the resin’s optical properties through light absorption, scattering, and refractive index mismatch. These effects dictate the spatial distribution of light energy within the resin, thereby influencing the curing depth and lateral cure dimensions. Increasing the concentration of 2D materials with light-absorbing characteristics may reduce the effective penetration depth of the projected light, whereas nanosheet agglomeration can lead to non-uniform light attenuation and localized variations during polymerization. Therefore, formulating DLP resins requires the simultaneous control of nanosheet concentration, dispersion stability, photoinitiator concentration, resin viscosity, exposure time, and layer thickness [67,68].

The relationship between 2D material concentration and DLP printability is particularly critical because DLP exposes the entire cross-sectional pattern simultaneously. Underexposure can result in incomplete polymerization and poor interlayer bonding, while overexposure may lead to excessive curing and the loss of fine geometric features. The presence of 2D nanosheets further modifies this process window by altering the resin’s light attenuation characteristics. Consequently, optimal exposure conditions for neat photocurable resins cannot be directly applied to formulations containing 2D materials; instead, exposure dosage and layer thickness must be recalibrated based on the optical properties and concentration of the incorporated nanosheets [59,67,68].

The feasibility of DLP printing for systems containing 2D materials has been demonstrated. Salas et al. developed a photocurable resin containing Ti_3_C_2_Tz MXene and demonstrated the capability to fabricate complex 3D composite structures with high printing fidelity using DLP technology. Subsequent annealing enhanced the electrical conductivity of the printed structures, thereby proving the potential of DLP for manufacturing structurally complex, conductive components [69]. This case also highlights a key characteristic of DLP printing based on 2D materials: there can be significant differences between the as-printed structure and the final functional material, as post-processing steps alter the polymer matrix, the chemical properties of the nanosheets, and the state of conductive connections [69].

DLP has also been employed to fabricate ceramic structures containing 2D materials. For instance, graphene oxide/hydroxyapatite composite ceramic scaffolds with controllable porous structures were prepared using DLP technology. Zhao et al. reported that the incorporation of a small amount of graphene oxide (GO) altered the light-scattering behavior of the ceramic slurry, helping to maintain the designed pore geometry during DLP, while subsequent debinding and sintering steps formed the final ceramic structure [70]. This study demonstrates that DLP serves not only as a direct fabrication technique for polymer-2D material composites but also as an intermediate shaping method for ceramic systems containing 2D materials.

Compared to SLA, the primary advantage of DLP lies in its parallel layer-by-layer exposure mechanism, which significantly enhances printing efficiency—particularly for structures with large cross-sectional areas. In contrast, conventional SLA utilizes a raster scanning mechanism, often requiring prolonged exposure times when fabricating large-area or tall structures. However, the achievable feature size in DLP depends heavily on the optical projection system, pixel size, light scattering, curing kinetics, and resin formulation [59,67]. Furthermore, increasing the concentration of 2D nanosheets to enhance electrical, thermal, or mechanical properties may simultaneously reduce the light penetration depth and narrow the printable process window. Consequently, a major challenge in applying DLP to 2D material fabrication is striking a balance between functional material loading and optical processability [68].

### 3.4. Droplet-Based and Other Techniques

Droplet-based printing technologies enable the controlled deposition of functional inks at predetermined locations. Depending on the materials used, curing mechanisms, and deposition sequences, these techniques can be employed either to construct three-dimensional objects or to form functional layer patterns on substrates [71]. A distinction must be made between these two types of applications: 3D manufacturing involves constructing structures with specific volumetric geometries according to a design, whereas planar printing primarily entails forming thin films, electrodes, or device layers on a substrate. Simply increasing film thickness through repeated deposition is not equivalent to fabricating a designed 3D architecture.

Inkjet printing is primarily categorized into two modes: continuous jetting and drop-on-demand (DOD) jetting. DOD printing is widely used for 2D material inks due to its ability to selectively position droplets and its relatively low material consumption. Such applications require precise control over ink viscosity, dispersion stability, substrate wettability, and solvent evaporation rates to achieve continuous and uniform functional layers.

Related techniques, including electrohydrodynamic jet (E-jet) printing and laser-induced forward transfer (LIFT), also enable the localized deposition or transfer of 2D materials [72]. Their classification depends on the actual manufacturing geometry achieved, rather than solely on the name of the technique.

The aerosol jet printing technique reported by Hu et al. [73]. represents a recent example of achieving true 3D manufacturing; this method utilizes additive-free, aqueous Ti_3_C_2_T_x_ MXene ink to construct self-supporting, free-form microstructures without the need for external support. Solvent evaporation during aerosol transport and deposition, combined with inter-nanosheet interactions, facilitates the formation of freestanding pillars and interconnected 3D networks. The researchers applied these printed structures to microsupercapacitor devices, demonstrating that aerosol-based deposition can overcome the limitations of planar patterning to enable the fabrication of functional 3D MXene structures.

The suitability of 2D materials for printing depends in part on their surface chemistry and dispersion behavior—factors that influence solvent compatibility, interactions between nanosheets, and formulation stability. Table 1 summarizes the surface characteristics and dispersion-related considerations for representative 2D materials.

### 3.5. Preparation of 2D Materials as Feedstocks for 3D Printing Inks

The preparation of 2D materials as raw feedstocks for 3D printing inks is a critical upstream process that determines the quality, scalability, and performance of the final printed structures. These methods aim to produce high-purity, defect-controlled nanosheets with uniform lateral sizes and thicknesses, while ensuring compatibility with solvent systems for stable dispersions [18]. Broadly categorized into top-down and bottom-up approaches, preparation techniques must minimize aggregation, oxidation, and structural damage to preserve intrinsic properties such as electrical conductivity and mechanical strength [2].

The influence of feedstock selection on printed structures was demonstrated by Freyman et al., who compared graphene oxide and reduced graphene oxide precursors for direct ink writing of carbon scaffolds. Following printing, freeze-drying, and thermal processing, the different precursors produced distinct pore structures and electrochemical responses. These findings connect precursor chemistry with structural evolution and functional performance, indicating that feedstock selection should consider its effects throughout the manufacturing process, in addition to dispersion and printability [74].

**Figure 3:**
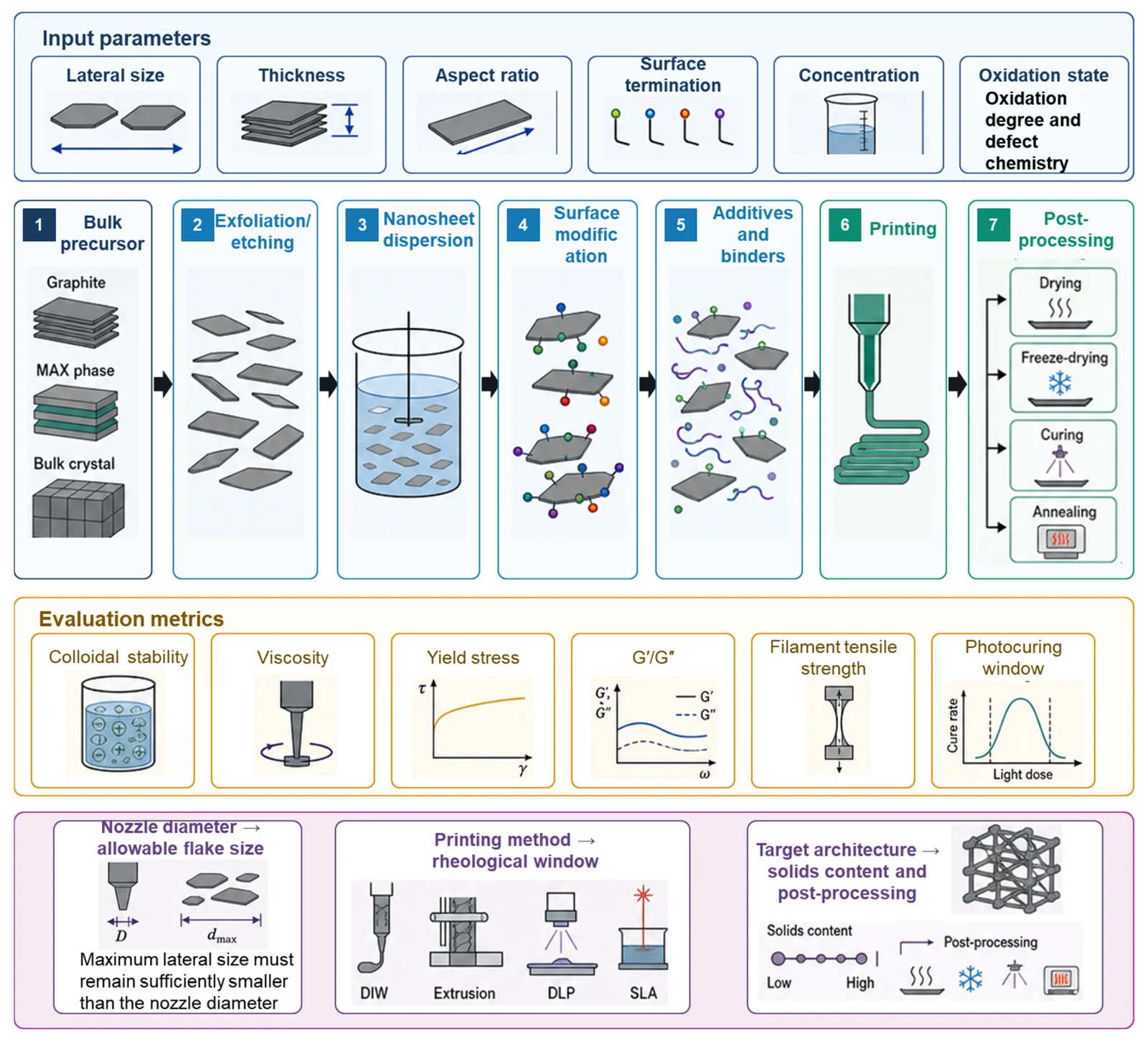
Feedstock preparation and printability evaluation for 2D-material-based additive manufacturing.

As summarized in Figure 3, feedstock preparation requires coordinated regulation of nanosheet dimensions, surface chemistry, dispersion state, additives, rheological or melt-flow behavior, and post-processing conditions.

Top-down methods predominate for scalable production. Liquid-phase exfoliation (LPE) uses ultrasonication or shear mixing to produce few-layer nanosheets from layered bulk materials. The resulting concentration and exfoliation yield depend strongly on the precursor, solvent, energy input, and post-exfoliation size-selection process [25]. Surfactants and polymers can improve colloidal stability through steric, electrostatic, or electrosteric interactions, thereby enabling higher solid concentrations in selected 2D-material dispersions [22,75]. For MXenes, selective etching of MAX phases using hydrofluoric acid (HF) or milder LiF/HCl mixtures produces multilayered stacks, followed by delamination via intercalants like tetrabutylammonium hydroxide (TBAOH) to obtain single/few-layer flakes with hydrophilic surfaces [23].This method ensures high aspect ratios and minimal defects, as evidenced by Raman spectroscopy [76].

Bottom-up synthesis offers precise control. Chemical vapor deposition (CVD) grows monolayer graphene or TMDs on substrates, followed by transfer and dispersion for ink formulation [24]. Epitaxial growth or solvothermal methods synthesize BP or *h*-BN, though scalability remains limited [77]. Post-preparation purification, such as centrifugation to remove unexfoliated particles, and functionalization enhance dispersibility and rheological tunability [78]. Recent advancements include green solvents and mechanochemical exfoliation for eco-friendly, high-throughput production [79].

The ink preparation process integrates these feedstocks into printable formulations. A typical workflow involves: (1) dispersion of exfoliated 2D materials in solvents via ultrasonication; (2) addition of binders or rheology modifiers for viscoelasticity; (3) homogenization and degassing to eliminate bubbles; and (4) rheological characterization. This culminates in printing-ready inks with tailored viscosities for specific techniques.

**Figure 4:**
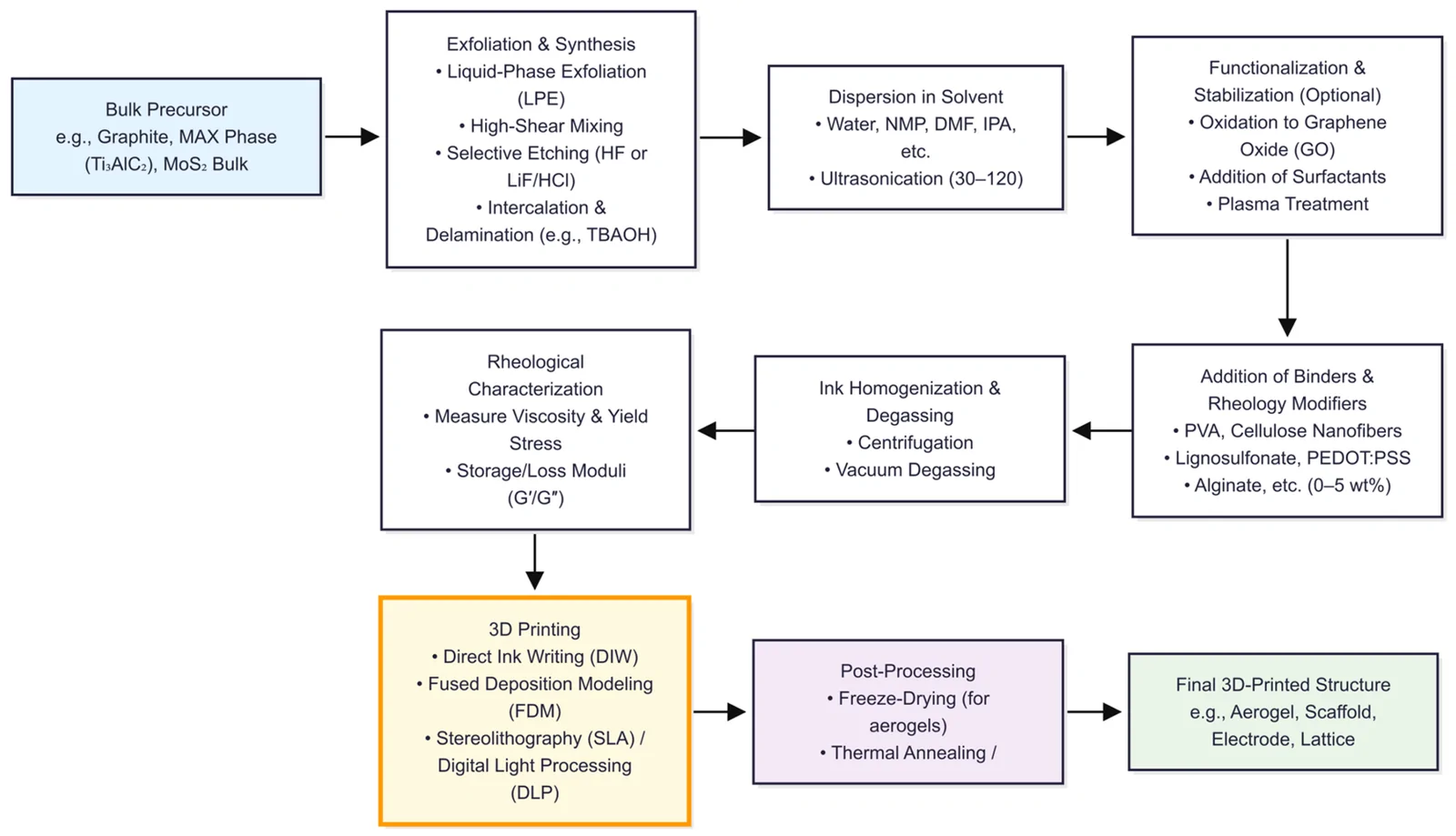
Process from 2D material preparation to printing.

For example, Figure 4 illustrates the sequential steps, from the preparation of two-dimensional materials to the printing process. [13,40].

This flowchart highlights key transitions: from raw material synthesis to ink optimization and final fabrication, ensuring process reproducibility. These compositions underscore the versatility of 2D inks, with viscosities tailored to printing modalities (e.g., high for DIW, low for inkjet) [40].

**Figure 5:**
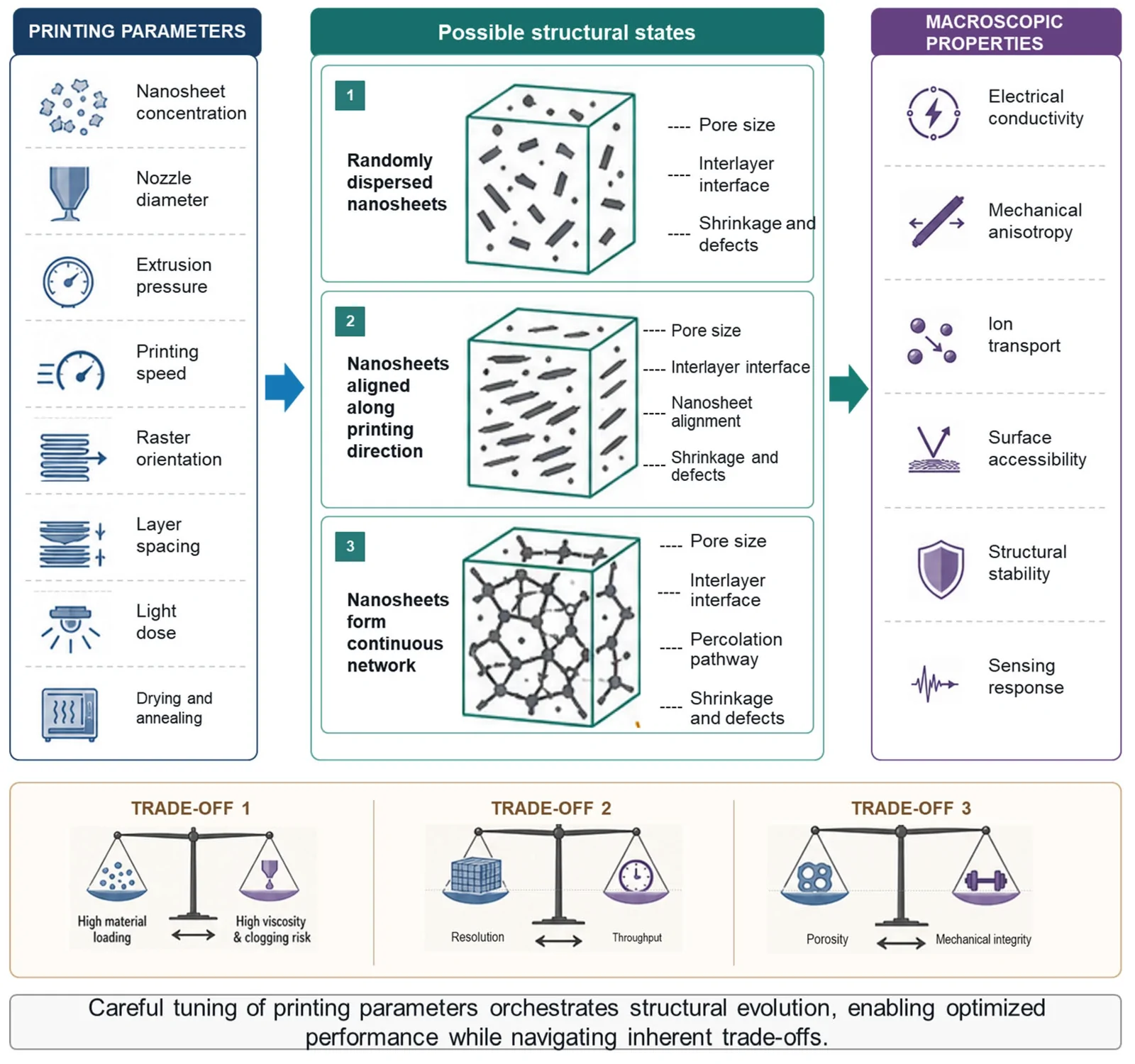
Process-induced structural features and structure–property relationships in 3D-printed 2D-material systems.

Beyond feedstock printability, the functional performance of printed structures is governed by process-induced changes in nanosheet orientation, pore architecture, interlayer interfaces, and percolation pathways, as summarized in Figure 5.

The rheological properties of the feedstocks influence their suitability for different printing methods. Table 2 summarizes reported viscosity values and measurement conditions for feedstocks containing representative 2D materials. Since viscosity depends on formulation, temperature, and testing conditions, these values should be interpreted in the context of their respective measurement conditions.

## 4. Application

The integration of two-dimensional (2D) materials—such as graphene, transition metal dichalcogenides (TMDs), MXenes, hexagonal boron nitride (*h*-BN), and black phosphorus (BP)—with 3D printing technologies has enabled the fabrication of complex, hierarchical structures that leverage their unique properties, including high conductivity, large surface area, mechanical flexibility, and biocompatibility [16]. This section reviews prominent applications of 3D-printed 2D materials in functional devices, including sensors, energy storage devices, biomedical applications and optoelectronic devices.

**Figure 6:**
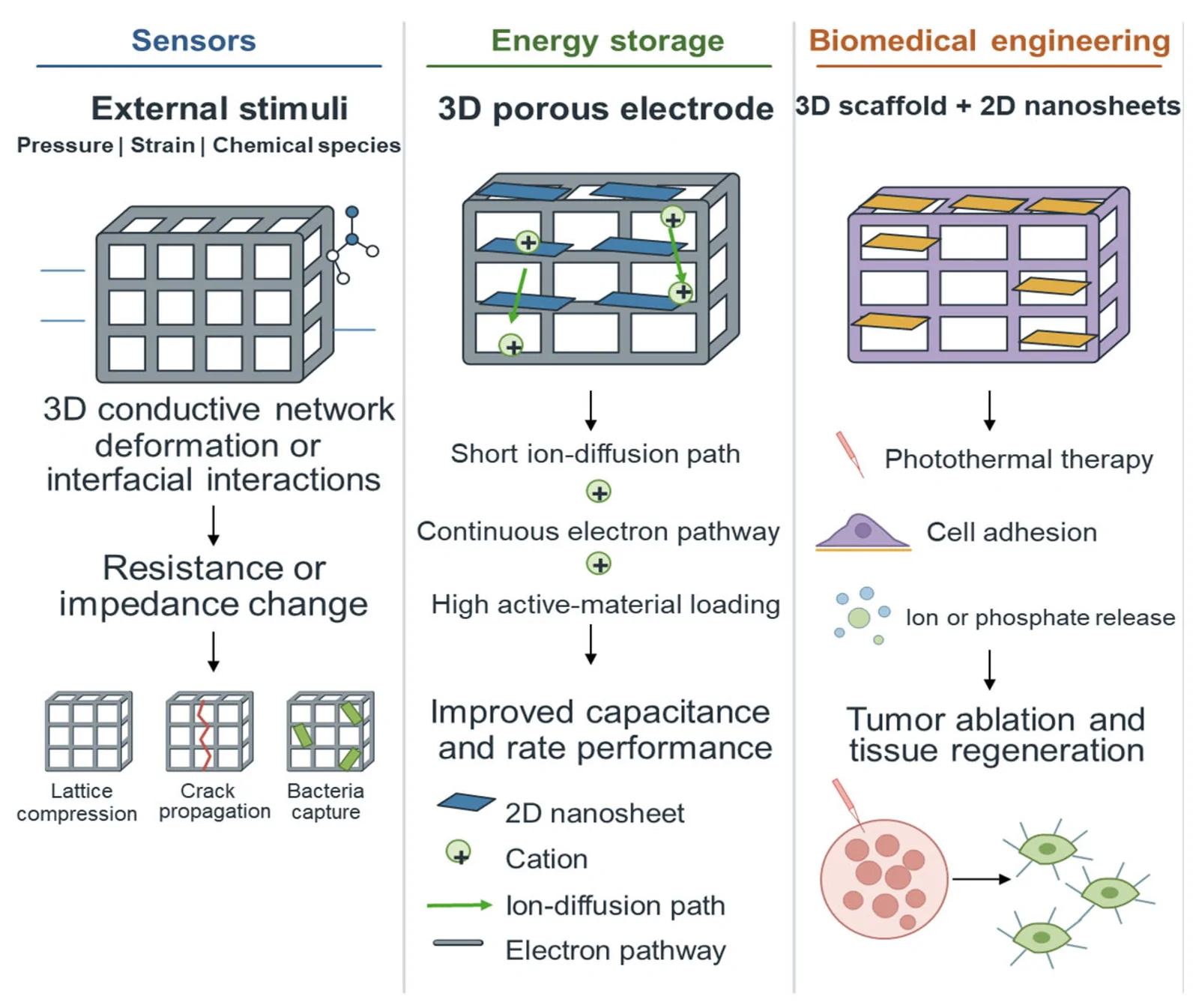
Functional mechanisms and representative applications of 3D-printed 2D materials.

Figure 6 summarizes the principal functional mechanisms through which 3D-printed 2D-material architectures contribute to sensing, energy storage, biomedical engineering.

Table 3 provides a quantitative overview of the electrical, thermal, and mechanical properties—as well as the applications—of typical 3D printing systems incorporating two-dimensional materials. Specific mechanical quantities and corresponding test conditions are listed to clarify the meaning of the reported values. When interpreting this data, material composition, printed structures, and test conditions should be considered collectively.

### 4.1. Sensors Devices

Two-dimensional materials (2D materials), such as graphene, transition metal dichalcogenides (TMDs), and MXenes, have demonstrated significant advantages in the sensor field due to their atomic-level thickness, ultra-high specific surface area, excellent electrical conductivity, mechanical flexibility, and chemical reactivity [103,104,105]. In recent years, these 3D-printed 2D material sensors have made significant progress in strain/pressure sensing, gas/humidity detection, electrochemical sensing, and multimodal intelligent sensing, opening new pathways for applications such as wearable electronics, environmental monitoring, and smart devices [105,106,107].

Lim et al. proposed a method for fabricating high-performance strain sensors using graphene-based Fused Deposition Modeling (FDM) 3D printing technology. They compared the performance of electrodes printed with graphene filaments in two different orientations. The results demonstrated that when the stacking direction was parallel to the printing direction (180°), the sensor exhibited higher sensitivity than the one printed using a zigzag pattern (45°); however, the 45° printed sensor showed lower stability and reliability due to higher electrical resistance under the same strain. To explain this phenomenon, the researchers conducted crack propagation simulations based on resistance data, and the simulation results aligned with measurements from mechanical experiments. The Gauge Factors (GF) for the 180° and 45° pattern sensors were 58 and 32, respectively, with both exhibiting linearity exceeding 90%. Furthermore, the sensor’s high stability was verified through 1000 bending cycles. Using this 3D-printed strain sensor, the researchers characterized its performance under top compression, lateral compression, and torsion conditions. The response curves indicate that the sensor holds potential for applications in the field of wearable devices [108].

Liu et al. fabricated a lightweight MXene/reduced graphene oxide (MX/rGO) pressure sensor using direct ink writing and freeze-drying techniques. The incorporation of octadecylamine enhanced the bonding between MXene and graphene oxide, significantly improving the gelation capability of inks containing low concentrations of 2D nanomaterials. Notably, owing to its 3D hierarchical lattice porous structure, the MX/rGO pressure sensor achieved high sensitivity (166.957 kPa^−1^), a wide sensing range (0.028–100 kPa), and excellent cyclic stability (10,500 cycles). These sensing capabilities enable the detection of various human activities, such as pulse, vocal cord vibrations, facial expressions, joint movements, and pressure distribution. This study opens up broad prospects for the development of customized flexible pressure sensors based on MXene-ink 3D printing technology [109].

MXene structures fabricated via direct ink writing (DIW) technology have seen their sensing applications expand into the realms of humidity monitoring and multifunctional operation. Xu et al. formulated a composite ink comprising MXene and carboxylated cellulose nanofibers, utilizing the cellulose component as a rheology modifier to meet the processing requirements of DIW [110]. The resulting printed grating structures integrate humidity sensing, electromagnetic interference shielding, and Joule heating capabilities. This example demonstrates how composite ink design can impart multiple functions to printed structures, thereby broadening the application prospects of 2D materials in multifunctional devices.

These studies reveal distinct relationships between fabrication strategies and sensing functionalities. Xu et al. further demonstrate that ink composition can support humidity sensing alongside shielding and heating functions, extending the discussion to multifunctional printed devices [110]. In the FDM-printed graphene strain sensors reported by Lim et al., altering the deposition pattern influences electrical and mechanical responses, highlighting the importance of scanning direction and crack evolution within the conductive composite [108]. In contrast, the DIW-printed MXene/rGO pressure sensors developed by Liu et al. rely on a hierarchical porous lattice structure that couples structural deformation with changes in conductive pathways [109]. Consequently, the reported gauge factors and pressure sensitivities reflect different sensing characteristics, making it impossible to directly rank their performance.

### 4.2. Energy Storage Devices

The exponential growth in portable electronics, electric vehicles, and renewable energy grids has precipitated a critical demand for advanced energy storage systems (ESSs) that simultaneously offer high energy density, high power density, and geometric conformability [111,112]. Two-dimensional materials, such as graphene, transition metal dichalcogenides (TMDs), and MXenes, exhibit significant advantages in energy storage devices (including lithium-ion batteries, sodium-ion batteries, zinc-ion batteries, and supercapacitors) due to their atomic-level thickness, ultra-high specific surface area, excellent electrical conductivity, superior mechanical flexibility, and rapid ion/electron transport properties [2,113].

#### 4.2.1. Supercapacitor

Supercapacitors (SCs), bridging the gap between conventional dielectric capacitors and batteries, occupy an irreplaceable role in portable electronics, regenerative braking systems, and high-pulse power applications due to their high power density, rapid charge/discharge rates, and exceptional cycle stability [114].

Recent studies have demonstrated the potential of 3D printing to integrate 2D-material-based supercapacitors with other functional components. Zhu et al. developed an additive-free, aqueous MXene ink with suitable rheological properties for the customized 3D printing of MXene-based micro-supercapacitors (M-SMSCs) on various substrates. The printed devices exhibited a voltage window of 1.8 V, an areal capacitance of approximately 4 F cm^−2^, and an areal energy density of 1772 μWh cm^−2^. Notably, these devices remained functional at low temperatures as extreme as −40 °C. Furthermore, the authors constructed a microsystem integrating an M-SMSC with a humidity sensor on a single substrate, demonstrating the potential of 3D printing technology for applications combining energy storage and sensing [115].

Cao et al. fabricated fully 3D-printed flexible micro-supercapacitors using a thixotropic ink composed of one-dimensional conductive cellulose and two-dimensional Ti_3_C_2_T_x_ MXene nanosheets. The resulting devices achieved an areal capacitance of 3.12 F cm^−2^ and an energy density of 1.25 mWh cm^−2^, while retaining 94% of their initial capacitance after 10,000 bending cycles. The study also demonstrated a self-powered sensing system capable of monitoring human motion, highlighting the potential of such printed devices in the fields of flexible and wearable electronics [116].

Studies by Zhu et al. and Cao et al. demonstrate complementary strategies for developing printable MXene-based supercapacitors. Zhu et al. utilized additive-free MXene ink to fabricate 3D-printed micro-supercapacitors with an expanded operating voltage window [115], whereas Cao et al. combined conductive cellulose with MXene to create a printable composite ink for flexible energy storage devices [116]. The former highlights the direct application of MXene as a printable electrode material, while the latter showcases a composite ink approach that balances electrochemical performance with mechanical flexibility. Consequently, performance evaluation should take into account the respective design objectives: capacitance and operating voltage are key metrics for energy storage performance, while capacitance retention under repeated bending is particularly critical for flexible applications. Collectively, these studies demonstrate the immense potential of MXene-based inks and composite formulations for fabricating flexible energy storage devices and integrating them with sensing functions.

Beyond MXene-based systems, Wang et al. combined photopolymerization-based 3D printing of graphene oxide precursors with computational structural optimization to fabricate ultra-thick graphene electrode architectures featuring an interpenetrating structure. This geometric design effectively addresses the challenges of ion and electron transport within thick electrodes, thereby enhancing active material utilization and achieving superior volumetric energy storage performance. The study extends the scope of research from printable ink compositions to device-level spatial design, demonstrating how 3D electrode architectures can boost the performance of graphene-based supercapacitors [117].

#### 4.2.2. Lithium Ion Battery

Two-dimensional (2D) materials—such as graphene, MXenes, and transition metal dichalcogenides (TMDs)—have surfaced as ideal building blocks for functional inks, owing to their unique shear-thinning rheology, exceptional in-plane conductivity, and mechanical flexibility to accommodate volume expansion. The synergistic integration of 3D printing’s structural tailorability with the electrochemical superiority of 2D materials paves a new avenue for developing next-generation LIBs featuring ultra-high areal capacity, fast-charging capabilities, and geometric conformability [118].

In the practical application of lithium-ion batteries (LIBs), the advantages of two-dimensional materials are primarily realized through two complementary design strategies: constructing electrode architectures to provide short ion-transport pathways and enable high active-material loading, and tailoring ink rheological properties to ensure reliable deposition and the formation of continuous conductive networks. Recent research has addressed both approaches, ranging from the design of hierarchical porous structures in composite electrodes to the formulation and viscoelastic tuning of 2D-material inks for extrusion-based printing. These studies demonstrate that the electrochemical performance of 3D-printed electrodes relies on the synergistic optimization of nanosheet composition, ink processability, and printed architecture, rather than solely on material selection.

Mwizerwa et al. fabricated a lithium-ion battery anode featuring a porous grid pattern using activated carbon/reduced graphene oxide composites via the direct ink writing technique [119]. The printed structure possesses hierarchical open pores, which shorten ion diffusion paths and enhance electrolyte permeability; simultaneously, the interconnected rGO network provides continuous electron transport channels and suppresses the agglomeration of activated carbon during the printing process. This AC@rGO anode retained a specific capacity of 756 mAhg^−1^ after 1000 cycles at a 1C rate and achieved an areal capacity of 27.09 mAhcm^−2^ with an active material loading of 40 mgcm^−2^. Furthermore, the authors validated the anode by assembling a full cell with a 3D-printed lithium iron phosphate (LiFePO_4_) cathode. This study demonstrates how the combination of a functional 2D nanosheet network and a well-designed 3D porous structure can facilitate ion and electron transport in high-loading composite electrodes.

Kumarov et al. developed a high-viscosity MXene electrode ink with optimal viscoelastic properties, suitable for fabricating lithium-ion battery prototypes via extrusion-based 3D printing. The E-MXene and E-D-MXene anodes demonstrated stable cycling performance, achieving specific capacities of 139.4 mAh·g^−1^ and 174.5 mAh·g^−1^, respectively, at a 0.2C rate. This single-layer, fully 3D-printed half-cell exhibited a high electrode mass loading of approximately 8.4 mg·cm^−2^. This research paves the way for the design and preparation of 3D-printable MXene-based composite anode inks, enabling the fabrication of precisely structured components, configurations, and systems with diverse functionalities and application scenarios [120].

Research by Mwizerwa et al. and Kumarov et al. demonstrates complementary strategies for developing 3D-printed lithium-ion battery anodes. In the AC@rGO system, direct ink writing (DIW) technology combines a hierarchically porous structure with an interconnected reduced graphene oxide (rGO) network, thereby enhancing electrolyte accessibility and electron transport performance even at high active material loadings [120]. In contrast, MXene-based research focuses on ink formulation and the modulation of viscoelastic properties to enable the extrusion printing of functional electrodes [121]. Collectively, these examples highlight the importance of balancing material composition, feedstock processability, and electrode structural design. Evaluating printed anodes requires considering electrochemical performance alongside manufacturing process requirements and the structural features that dictate transport characteristics.

### 4.3. Biomedical and Tissue Engineering

The rapid development of additive manufacturing (3D printing) technology has revolutionized the fields of biomedicine and tissue engineering, making it possible to manufacture biomimetic scaffolds with patient-specific anatomical structures and complex microenvironments [121]. However, current mainstream polymer-based bioinks often face bottlenecks such as insufficient mechanical strength, lack of electroactivity, and limited bio-induction capabilities, making it difficult to meet the needs of high-performance applications such as bone regeneration, nerve repair, and intelligent biosensing [122]. To overcome this limitation, two-dimensional (2D) nanomaterials, represented by graphene, transition metal carbon/nitrides (MXenes), and molybdenum disulfide (MoS_2_), are becoming key components of next-generation functional bioinks due to their ultra-high specific surface area, excellent mechanical properties, unique conductivity, and photothermal response characteristics [123]. Integrating these two-dimensional materials into 3D printing systems can not only significantly improve the rheological properties and printability of inks but also endow printed structures with multifunctional properties that mimic the natural extracellular matrix (ECM), thus showing great application potential in promoting tissue regeneration, drug delivery, and multimodal therapy integration [124].

By rationally integrating 2D Ti_3_C_2_ MXene with a 3D-printed bioactive glass (BG) scaffold, Pan et al. achieved the dual objectives of eliminating bone tumors via photothermal therapy and promoting bone tissue regeneration through a bioactive scaffold. Leveraging the excellent photothermal conversion properties of the 2D Ti_3_C_2_ MXene material, the composite scaffold enables bone tumor ablation driven by near-infrared light-induced photothermal therapy, successfully achieving complete tumor eradication in a xenograft animal model. Furthermore, the study demonstrated that the incorporation of 2D Ti_3_C_2_ MXene effectively accelerates the *in vivo* growth of new bone tissue on the composite BG scaffold. This Ti_3_C_2_ MXene composite scaffold, combining bone tumor ablation with bone tissue regeneration capabilities, offers a highly promising strategy for treating bone tumors and significantly expands the biomedical application potential of 2D MXenes in the field of tissue engineering—particularly for bone tumor therapy [125].

Graphene oxide (GO) has attracted much attention in the biomedical field due to its excellent properties—such as high specific surface area, ability to bind therapeutic functional molecules, solubility, high mechanical strength, and excellent thermal conductivity. L. González-Rodríguez et al. developed a PLA : CaP : GO three-dimensional printed scaffold for bone tissue engineering. Through in vitro (human osteoblast-like cells MG-63) and in vivo (zebrafish model) direct exposure experiments, the optimal GO dosage to ensure non-toxicity was determined. Furthermore, the effects of GO on immune cells were assessed using zebrafish microinjection experiments, and the gene expression levels of key biomarkers were quantitatively detected. The results showed that the tested GO (C:O ratio 2.14, oxidation rate 49.50%, major bond types: C–OH, C–O–C) at concentrations ≤ 0.25 mg mL^−1^ resulted in MG63 cell survival exceeding 70%, and concentrations ≤ 0.10 mg mL^−1^ were non-toxic to zebrafish embryos. Immunological assessments reinforced these results. Finally, a three-dimensionally printed PLA : CaP : GO scaffold was fabricated by combining an optimized dose (0.10 mg·mL^−1^) of GO with polylactic acid (PLA) and calcium phosphate (CaP). These findings provide strong validation for the future application of GO-enriched composite materials in the field of biomedical engineering [126].

Black phosphorus (BP) has attracted much attention in the field of biomedical applications due to its unique mechanical and electrochemical properties. This study investigated the synergistic effect of these two types of two-dimensional materials on osteogenic activity in bone tissue engineering. First, BP was encapsulated in negatively charged GO nanosheets, which were then co-adsorbed onto the surface of a positively charged poly(propylene fumarate) three-dimensional (3D) scaffold. The increased surface area provided by the GO nanosheets enhanced cell adhesion in the initial stage. Furthermore, the slow oxidation process of the BP nanosheets encapsulated within the GO layer continuously released phosphate—a key osteoblast differentiation promoter designed to stimulate cell differentiation towards new bone formation. Using three-dimensional confocal imaging, a unique interaction between cells and BP nanosheets was observed: cells exhibited a stretched morphology, microfilament structures formed around the BP nanosheets, and cell proliferation capacity was significantly better than that of scaffolds containing only a single two-dimensional material. Moreover, the biomineralization and osteogenic markers of the three-dimensional scaffold were enhanced in scaffolds simultaneously integrating BP and GO nanosheets. All results indicate that integrating two-dimensional BP and GO materials into a three-dimensional tissue scaffold can effectively synergistically promote cell proliferation and osteogenic processes [127].

These studies on scaffolds vary in terms of their intended therapeutic functions and the evidence used for evaluation. Pan et al. combined MXene-mediated photothermal tumor therapy with the regenerative capabilities of 3D-printed bioactive glass scaffolds [125]. González-Rodríguez et al. focused on determining optimal graphene oxide (GO) exposure conditions prior to incorporating GO into poly(lactic acid)/calcium phosphate (PLA/CaP) scaffolds, subsequently assessing biosafety through experiments involving cells and zebrafish [126]. Liu et al. investigated the synergistic effects of GO and black phosphorus (BP) within printed scaffolds, linking enhanced cell adhesion to phosphate release during BP oxidation [127]. These functional advantages stem from diverse mechanisms, including photothermal conversion, cell-material interactions, and controlled degradation. A comparative analysis of these studies indicates that dosage, degradation behavior, and the extent of biological validation must all be considered when evaluating therapeutic functions.

### 4.4. Optoelectronic Devices and Photodetectors: Relevant Planar Printing Methods

Planar printing of optoelectronic devices offers a complementary perspective on the processing and integration of 2D materials. The examples discussed in this subsection focus on the deposition of functional thin films and electrodes onto substrates, rather than the construction of specific 3D structures. These cases are included because their requirements regarding ink formulation, film-forming processes, and interface control provide valuable insights for the overall development of printed 2D material devices.

Solution-based printing techniques—particularly inkjet printing—enable low-cost, scalable, and patterned deposition on a variety of flexible substrates [128]. Depending on their composition and electronic properties, 2D materials such as graphene, molybdenum disulfide (MoS_2_), and MXenes can serve complementary roles in photodetectors, including charge transport, light absorption, and electrode formation [129]. However, translating these properties into functional printed devices requires simultaneous control over dispersion stability, ink viscosity, substrate wettability, and interfacial compatibility. For instance, research has demonstrated the fabrication of graphene/perovskite/graphene heterostructures using an all-inkjet printing process, as well as the printing of MoS_2_/graphene heterostructures on flexible substrates [130,131].

These examples illustrate different strategies for integrating photosensitive materials with conductive contact layers. In graphene/perovskite/graphene devices, graphene electrodes encapsulate the perovskite photosensitive layer, whereas MoS_2_/graphene devices combine a 2D semiconductor with graphene contact layers.

Table 4 summarizes the relationships among printing methods, 3D structures, and functional effects in the aforementioned application areas, as well as their respective primary limitations.

## 5. Challenges and Limitations

Although 3D printing of two-dimensional (2D) materials has shown remarkable promise, several inherent challenges and limitations persist, stemming from material properties, processing constraints, and application-specific requirements. These issues must be addressed to achieve broader adoption. This section discusses key limitations: materials stability, printing challenges, toxicity and biocompatibility, ink diversity, the contradiction between resolution and scale (balancing macroscopic large sizes with microscopic high precision), and shrinkage in macroscopic dimensions.

### 5.1. Material Stability

Many 2D materials exhibit poor stability during printing and post-processing, leading to degradation that compromises performance. MXenes are highly susceptible to oxidation in aqueous environments, resulting in rapid loss of conductivity and structural integrity [6]. Black phosphorus (BP) degrades quickly in air, with oxidation occurring within hours, necessitating inert conditions for handling [13]. Graphene oxide (GO) dispersions can restack or introduce defects during drying, increasing the Raman I_D_/I_G_ ratio significantly [16]. TMDs such as MoS_2_ can undergo phase transformations under certain external stimuli, which may affect their electronic and structural properties [9]. Hexagonal boron nitride (*h*-BN) is relatively stable but suffers from aggregation in inks [132]. These stability issues limit shelf life and reproducibility.

Recent research has explored processing strategies aimed at enhancing the structural stability of printed MXene structures. Osman et al. fabricated MXene/carbon aerogels by combining Direct Ink Writing (DIW) of MXene/cellulose nanofiber ink with freeze-drying and controlled annealing processes [133]. The carbonization of the cellulose component and the formation of Ti–O–C interfacial bonds enhanced the material’s stability during long-term storage in air and exposure to harsh environments. These results highlight the potential to improve the durability of printed structures through the synergistic design of ink formulations and post-processing techniques, while also underscoring the importance of precisely controlling annealing conditions.

### 5.2. Printing Challenges

Printing 2D materials is complicated by rheological incompatibilities and process sensitivities. High-viscosity inks often cause nozzle clogging in extrusion-based methods, while low-viscosity requirements for inkjet printing restrict material concentrations [40]. Light-based techniques like SLA struggle with light scattering from 2D flakes, reducing curing efficiency [72]. High temperatures in FDM degrade sensitive materials like BP [105,134]. Shear forces in direct ink writing (DIW) can misalign flakes or induce defects [7]. Overall, these challenges constrain print speed, fidelity, and material choice.

Table 5 summarizes these challenges, the materials involved, their potential impacts, and common mitigation strategies.

### 5.3. Toxicity and Biocompatibility

Toxicity remains a major concern, especially for biomedical applications. GO and rGO can generate reactive oxygen species (ROS), at relatively high GO concentrations, cell viability decreases [13]. MXenes show dose-dependent cytotoxicity, though generally lower than graphene [6]. BP induces oxidative stress and potential organ damage [41]. TMDs like MoS_2_ trigger mild proinflammatory responses [135]. *h*-BN is less toxic but can disrupt membranes at high doses [136]. Biocompatibility varies with size, surface chemistry, and dose; functionalization often mitigates risks but requires careful validation per ISO standards [13].

### 5.4. Ink Diversity

Ink formulations are limited in diversity, as pristine 2D materials rarely exhibit ideal rheology without additives. Most inks require polymers or surfactants, compromising purity and properties [40]. Aqueous systems dominate for MXenes, restricting solvent options [6]. TMDs and BP need stabilizers, reducing available compositions [9]. This lack of diversity hampers multi-material printing and application-specific tailoring [72].

### 5.5. Resolution and Scale Contradictions

A fundamental trade-off exists between achieving high printing resolution for precision applications and scaling fabricated structures to macroscopic dimensions. High-resolution techniques such as inkjet printing and stereolithography (SLA) can achieve feature sizes below 50 μm; however, they are typically constrained to small build volumes due to inherently slow deposition rates or limited light penetration depth. In contrast, extrusion-based printing methods enable the fabrication of larger and thicker architectures but generally sacrifice spatial resolution, as the minimum feature size is governed by nozzle diameter and complex flow dynamics [7,44]. This resolution–scale trade-off poses significant challenges for applications that demand both fine structural detail (e.g., sensors) and large device dimensions (e.g., energy storage electrodes or scaffolds), often leading to compromised functional performance upon scale-up [11].

### 5.6. Macroscopic Dimensions Shrinkage

Shrinkage during drying or post-processing remains a major source of dimensional inaccuracy and residual stress in large-scale printed structures based on two-dimensional (2D) materials. For porous assemblies such as graphene oxide (GO) and MXene aerogels, substantial volumetric contraction during solvent removal has been widely reported, frequently leading to cracking, warping, or loss of structural integrity in macroscopic constructs [7]. Thermally induced gradients during fused deposition modeling (FDM) further exacerbate anisotropic contraction in 2D material-reinforced composites, particularly for transition metal dichalcogenide (TMD)-based systems [14]. Similarly, printed architectures incorporating air-sensitive or highly anisotropic materials, such as black phosphorus (BP) and hexagonal boron nitride (*h*-BN), often exhibit nonuniform shrinkage during drying or post-treatment, resulting in distorted final geometries [41]. Although strategies such as controlled drying, post-curing, or the introduction of rheology-modifying additives can mitigate shrinkage-related defects, they inevitably increase processing time, system complexity, and overall manufacturing cost [7].

**Figure 7:**
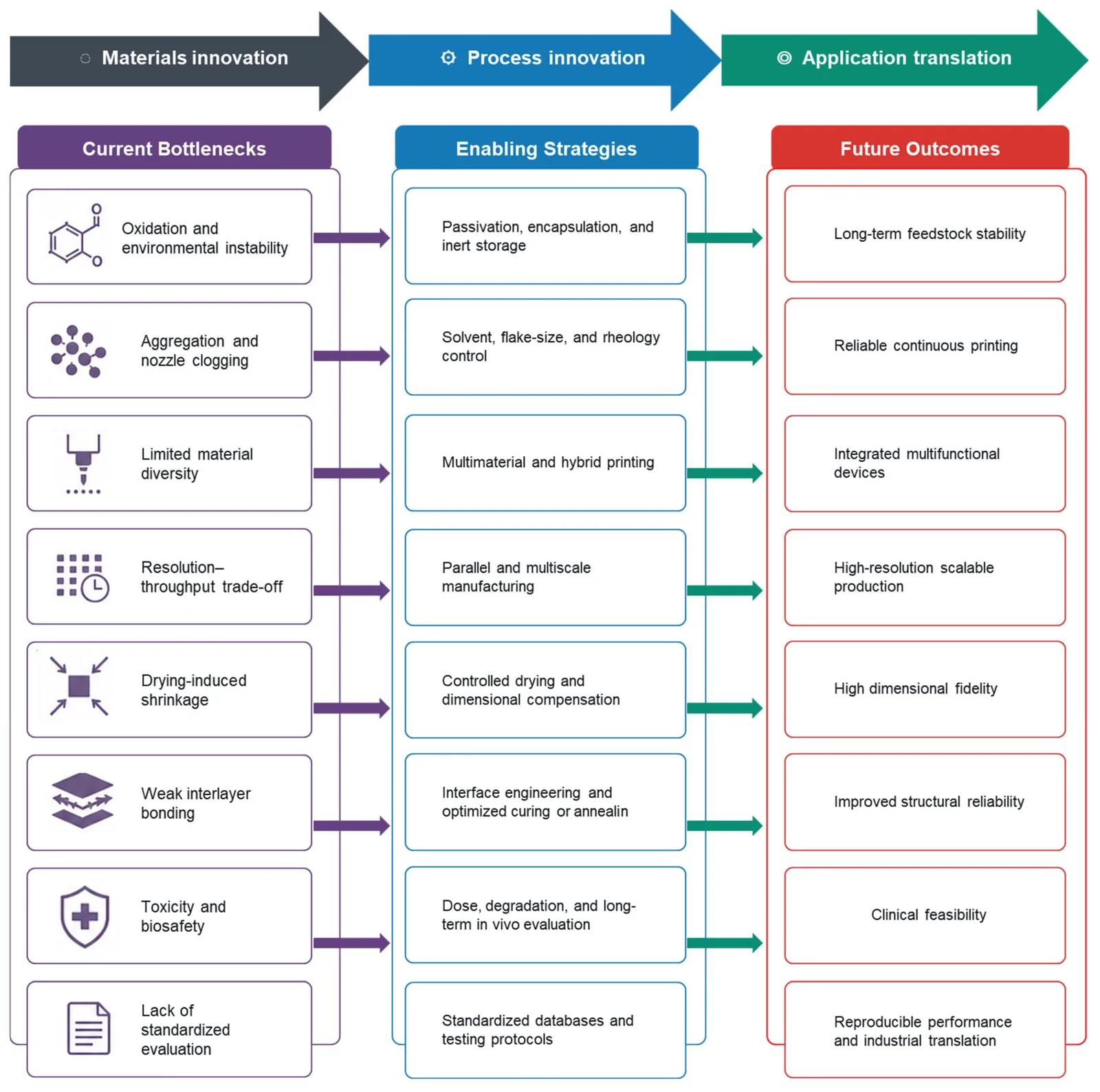
Current bottlenecks, enabling strategies, and expected outcomes for 3D printing of 2D materials.

Building on the limitations discussed above, Figure 7 links the principal material, process, and translational bottlenecks with corresponding enabling strategies and expected outcomes.

## 6. Conclusions and Future Perspectives

This review summarizes recent advances in the printing of two-dimensional (2D) materials and their integration into functional devices, focusing on the fabrication of 3D structures using nanosheet-based inks and composite feedstocks. These methods offer opportunities to address challenges associated with macroscopic assembly and device integration. However, realizing their full potential depends on material composition, printing conditions, structural design, and post-processing techniques. Research on planar printing is also discussed as a complementary approach for functional thin-film fabrication and device integration.

(1) Studies indicate that assembling graphene, MXenes, and other 2D materials into specific 3D structures can increase the effective contact area of nanosheets, facilitate charge or mass transport, and tune mechanical response characteristics. However, these performance enhancements are not universal, nor are they necessarily achievable simultaneously. Factors such as material restacking, interlayer contact resistance, structural defects, and deformation during drying can limit the advantages offered by structural design.
(2) The various printing techniques discussed possess distinct processing characteristics and limitations. Direct Ink Writing (DIW) relies heavily on ink rheology and shape-retention capabilities; Fused Deposition Modeling (FDM) requires materials with suitable melt flow properties, feedstock stability, and interlayer adhesion; and Stereolithography (SLA) and Digital Light Processing (DLP) technologies necessitate precise control over light attenuation and curing behavior in nanosheet-loaded formulations.
(3) The research reviewed demonstrates the potential of these materials—under specific experimental conditions—for applications in sensing, supercapacitors, battery electrodes, and biomedical scaffolds. However, direct comparisons between studies are limited by variations in composition, device geometry, performance normalization standards, and testing protocols.
(4) Significant challenges remain in the field, including material stability, a limited range of feedstocks, trade-offs between resolution and build size, post-processing shrinkage, and incomplete biosafety assessments. Overall, 3D printing technology for 2D materials is still in a stage of rapid development, and further systematization and standardization of both fundamental research and application exploration are urgently needed.

## Figures and Tables

**Table 1:** Comparison of the surface chemistry, dispersion-related behavior and key limitations of representative 2D materials.

2D Material	Surface Chemistry	Dispersion-Related Behavior	Key Limitation
Graphene	hydrophobic	limited aqueous dispersibility; susceptibility to aggregation and restacking	dispersion
GO	hydrophilic	generally dispersible in water; dispersion depends on pH, ionic strength, and sheet characteristics	reduction/shrinkage
MXene	hydrophilic	dispersions are compatible with water; stability depends on solution conditions and oxidation	oxidation
*h*-BN	relatively inert	pristine nanosheets may show limited compatibility with aqueous or polymer media	dispersion + light scattering
BP	oxidation sensitive	dispersion and stability depend strongly on solvent and protection conditions	environmental instability

**Table 2:** Advances in the Rheological Properties of 3D-Printed Two-Dimensional Materials.

2D Material	Component	Printing Method	Conditions	Viscosity, η (Pa·s)	Ref.
MXene	GelMA-TCP-Alg-0.3 mg ml^−1^ MXene	DIW	Temperature: 20 °C; Shear rate: 0.1 s^−1^	1000	[80]
MXene	TOCNF/MXene −50%	DIW	Shear rate: 0.1 s^−1^	10	[81]
Graphene	0.40%CNFs-6.40%Gelatin −1.60%Gellan Gum −0.50%COOH-GO	ME-3DP	Shear rate: 0.1 s^−1^	1000	[82]
Graphene	GNP/SiC_p_/PCS	DIW	Shear rate: 1 r/min	500	[83]
GO	GO/Glycerol	DIW	Shear rate: 0.1 s^−1^	10^4^	[84]
rGO	rGO/Co30	DIW	Circular frequency: 0.1 rad/s	10^5^	[85]
rGO	Carbopol hydrogel/rGO	DIW	Temperature: 25 °C; Shear rate: 0.1 s^−1^	10^4^	[86]
rGO	rGO/PCL/Mel	DIW	Shear rate: 0.1 s^−1^	10^3^	[87]
MoS_2_	PVDF-MoS_2_	DIW	Shear rate: 10 s^−1^	10^5^	[88]
MoS_2_	MoS_2_/GO	DIW	Shear rate: 0.1 s^−1^	10^5^	[89]
BN	BN/Polyacrylic Hydrogel	DIW	Temperature: 25 °C; Shear rate: 0.1 s^−1^	10^4^	[90]
BN	BN/CNT/PEEK	FDM	Temperature: 360 °C; Shear rate: 0.1 s^−1^	10^6^	[91]
BN	BN/Flex 63A	VP	Temperature: 25 °C; Shear rate: 10 s^−1^	5.36	[92]
BN	BC/PVA/BN	DIW	—	0.083	[93]

**Table 3:** Research Progress on the Properties and Applications of 3D-Printed Two-Dimensional Materials.

2D Material	Printing Method	Electrical Conductivity, σ (S/m)	Thermal Conductivity, k (W/(m·K))	Mechanical Testing Conditions	Mechanical Property	Value (MPa)	Application	Ref.
MXene	AJP	1.1 × 10^4^	—	—	—	—	Cardiac tissue engineering	[94]
MXene	DIW	—	—	Strain: 50% Compression speed: 1 mm/min Force sensor range: 100 N	Compressive stress	0.1	Biomedical applications	[80]
MXene	DIW	18.87	—	—	—	—	Flexible electrode	[95]
MXene	DIW	211	—	Loading rate: 1 mm/min	Tensile strength	75.6	Wearable heating textiles	[81]
Graphene	DIW	705.6	—	—	—	—	Piezoresistive pressure sensor	[96]
Graphene	FDM	0.11	—	Room temperature	Tensile strength	20	Strain sensing	[97]
Graphene	ME-3DP	—	—	Compression rate: 2 mm/min	Compressive strength	0.299	Bone tissue engineering	[82]
Graphene	DIW	670	—	50 mm cubic specimen	Compressive strength	4.3	Graphene/SiC composites	[83]
GO	ME-3DP	43.47	—	Strain:1.75%	Compressive stress	30	Thermomechanical sensing	[98]
GO	DLP	4.00 × 10^−7^	—	—	—	—	3D composites	[99]
GO	DIW	2073	—	Strain: 90%	Compressive stress	62.7	Tissue-engineered scaffolds	[84]
rGO	DIW	96	—	Strain: 80%	Compressive stress	1.7	Wearable energy devices	[85]
rGO	FDM	476	—	Tensile speed: 50 mm/min	Tensile strength	64	Organic electronics	[100]
rGO	DIW	863.7	—	Loading rate: 2 mm/min	Compressive strength	3.23	3D graphene assemblies	[86]
rGO	DIW	0.109	—	—	—	—	Peripheral nerve regeneration	[87]
MoS_2_	DIW	1	—	—	Tensile stress	0.0275	Healthcare monitoring	[101]
*h*-BN	DIW	—	0.8808	—	—	—	Microelectronic devices	[90]
*h*-BN	FDM	—	1.77	—	—	—	Advanced packaging materials	[91]
*h*-BN	VP	—	0.8	Temperature: 23 °C; Test load: 10 kN; Tensile speed: 10 mm/min	Tensile strength	2.2	Wearable systems	[92]
*h*-BN	DIW	—	—	—	Tensile strength	0.1	Bone tissue engineering	[93]
BP	DIW	7.55 × 10^4^	—	—	—	—	Wearable electronics	[102]

**Table 4:** Structure–processing–property relationships in 3D-printed 2D-material systems for different applications.

Application	2D Material	Printing Method	3D Architecture	Key Structural Effect	Main Performance Metric	Major Limitation
Strain sensing	Graphene	FDM	Layered/raster	Crack-controlled conductive network	GF	Anisotropy
Pressure sensing	MXene/rGO	DIW	Hierarchical lattice	Large deformation + conductive pathways	Sensitivity	Structural instability
Supercapacitor	MXene/graphene	IJP/DIW	Porous electrode	Improved ion accessibility	Areal capacitance	Restacking
Bone regeneration	GO/BP	3D printing	Porous scaffold	Cell adhesion + mass transport	Cell viability/osteogenesis	Long-term safety
Thermal management	*h*-BN	DIW/FDM/VP	3D network	Heat-transfer pathway	Thermal conductivity	Filler aggregation

**Table 5:** Common Printing Challenges for 2D Materials.

Challenge	Affected Materials	Impact	Typical Mitigation
Nozzle Clogging	GO, MXene	Interrupted flow, uneven deposition	Rheology modifiers
Light Scattering	TMDs, Graphene	Poor curing in SLA/DLP	Low loadings or composites
Thermal Degradation	BP, TMDs	Property loss in FDM	Post-print coating
Shear-Induced Defects	All	Misalignment, phase changes	Optimized nozzle design

## Data Availability

No new data were created or analyzed in this study. Data sharing is not applicable to this article.
